# Human Immunodeficiency Virus Type-1 Genetic Diversity and Drugs Resistance Mutations among People Living with HIV in Karachi, Pakistan

**DOI:** 10.3390/v16060962

**Published:** 2024-06-14

**Authors:** Abdur Rashid, Li Kang, Feng Yi, Qingfei Chu, Sharaf Ali Shah, Syed Faisal Mahmood, Yimam Getaneh, Min Wei, Song Chang, Syed Hani Abidi, Yiming Shao

**Affiliations:** 1 School of Medicine, Nankai University, Tianjin 300071, China; rasheed@mail.nankai.edu.cn (A.R.); weimin@nankai.edu.cn (M.W.); 2 National Key Laboratory of Intelligent Tracking and Forecasting for Infectious Diseases, Chinese Center for Disease Control and Prevention, Beijing 102206, China; lk201720073@outlook.com (L.K.); 22018100@zju.edu.cn (Q.C.); songchang723@chinaaids.cn (S.C.); 3 College of Life Sciences, Nankai University, Tianjin 300071, China; 4 State Key Laboratory for Diagnosis and Treatment of Infectious Diseases, National Clinical Research Center for Infectious Diseases, Collaborative Innovation Center for Diagnosis and Treatment of Infectious Diseases, The First Affiliated Hospital, School of Medicine, Zhejiang University, Hangzhou 310003, China; 12018626@zju.edu.cn; 5 Bridge Consultants Foundation, Karachi 75100, Pakistan; drsharafshah@yahoo.com; 6 Department of Medicine, Aga Khan University, Karachi 74800, Pakistan; faisal.mahmood@aku.edu; 7 Ethiopian Public Health Institute, Addis Ababa P.O. Box 1242, Ethiopia; 8 Department of Biological and Biomedical Sciences, Aga Khan University, Karachi 74800, Pakistan; 9 Department of Biomedical Sciences, School of Medicine, Nazarbayev University, Astana 010000, Kazakhstan; 10 Changping Laboratory, Yard 28, Science Park Road, Changping District, Beijing 102206, China

**Keywords:** HIV-1, genetic diversity, phylogeny, antiretroviral therapy, drug resistance, Karachi, Pakistan

## Abstract

The human immunodeficiency virus type-1 epidemic in Pakistan has significantly increased over the last two decades. In Karachi, Pakistan, there is a lack of updated information on the complexity of HIV-1 genetic diversity and the burden of drug resistance mutations (DRMs) that can contribute to ART failure and poor treatment outcomes. This study aimed to determine HIV-1 genetic diversity and identify drug-resistance mutations among people living with HIV in Karachi. A total of 364 HIV-positive individuals, with a median age of 36 years, were enrolled in the study. The HIV-1 partial *pol* gene was successfully sequenced from 268 individuals. The sequences were used to generate phylogenetic trees to determine clade diversity and also to assess the burden of DRMs. Based on the partial *pol* sequences, 13 distinct HIV-1 subtypes and recombinant forms were identified. Subtype A1 was the most common clade (40%), followed by CRF02_AG (33.2%). Acquired DRMs were found in 30.6% of the ART-experienced patients, of whom 70.7%, 20.7%, and 8.5% were associated with resistance to NNRTIs, NRTIs, and PIs, respectively. Transmitted DRMs were found in 5.6% of the ART-naïve patients, of whom 93% were associated with resistance against NNRTIs and 7% to PIs. The high prevalence of DRMs in ART-experienced patients poses significant challenges to the long-term benefits and sustainability of the ART program. This study emphasizes the importance of continuous HIV molecular epidemiology and drug resistance surveillance to support evidence-based HIV prevention, precise ART, and targeted AIDS care.

## 1. Introduction

Globally, antiretroviral therapy has helped reduce the rates of mortality and morbidity associated with HIV-1 infection. However, the emergence of HIV drug resistance (HIVDR) remains a threat to the effectiveness of antiretroviral therapy. Over the past decade, significant efforts have been made by the UNAIDS to achieve 95-95-95 goals, which aimed that by the end of 2022, 33.6 million people living with HIV (PLHIV) knew their HIV status, 29.8 million PLHIV were accessing antiretroviral therapy, and 27.7 million PLHIV were virally suppressed [1]. 

According to WHO reports, the prevalence of DRM increased from 6.8% to 10% among antiretroviral therapy (ART) initiators in developing countries between 2010 and 2017 [2]. HIV drug resistance has been reported for all major antiretroviral (ARV) drugs, potentially resulting in delayed viral suppression or treatment failure in individuals initiating ART [3]. HIVDR surveillance is conducted in most countries, enabling the monitoring of HIV susceptibility and tracing of virus mobility [4,5]. Understanding HIVDR at the population level can aid countries in selecting the optimal first-line ARV, mainly when HIVDR testing is not conducted prior to ART initiation [6].

In Pakistan, the HIV/AIDS epidemic has increased significantly over the last two decades [7]. Pakistan experienced a 78.5% increase in new HIV infections from 2010 to 2019, with an annual incidence of 20,000 HIV infections [8]. As of December 2023, an estimated 270,000 people are living with HIV (PLHIV). Pakistan’s progress toward 95-95-95 goals is lagging behind, as only 24.5% of PLHIV in Pakistan are aware of their HIV status, and just 16.2% are on ART. At the same time, data on viral suppression are sparsely available http://www.nacp.gov.pk (accessed on 1 March 2024). UNAIDS country progress report on AIDS and a recent study estimated the viral suppression rates to be 23% and 40%, respectively [9,10].

Recent surveillance reports showed that the epidemic is concentrated among the following HIV key population groups, which have consistently demonstrated high prevalence; for example, 38.4% in people who inject drugs PWID, 7.5% in transgender sex workers, and 5.6% in male sex workers [11].

In Pakistan, with rising new HIV cases, there are serious challenges related to access and adherence to ART. In 2004, the National AIDS Control Programme (NACP) initiated the ART program in all four provinces with the support of the Global Fund. Currently, there are 89 ART centers located in different parts of the country that provide antiretrovirals free of cost to all HIV patients [12]. The primary ART regimen for PLHIV consists of two nucleoside reverse transcriptase inhibitors (NRTIs), specifically lamivudine (3TC) and tenofovir, combined with the integrase inhibitor dolutegravir (DTG) while abacavir (ABC) and zidovudine (AZT) may also be included [13]. ART in Pakistan is typically initiated without conducting baseline or periodic sequencing, increasing the likelihood that individuals may receive ineffective treatment [13]. There are limited data on HIV-1 drug resistance mutations among Pakistani PLHIV. Our previous report on HIV DRM analysis from Sindh, Pakistan, showed the presence of DRM among 6.5% of ART-naïve individuals and 36.4% of ART-experienced individuals [14]. 

Karachi is the capital city of Sindh and the largest financial city in Pakistan. It is located on the coast of the Arabian Sea and has a population of more than 17 million. The first cases of HIV-1 were reported in Karachi in 1987 [15]. According to the Sindh province HIV/AIDS epidemiology database, in 2021, Karachi had the highest number of HIV-1 cases (n = 6768), where 40% of cases were reported in Karachi’s central district alone [16]. The HIV-1 epidemic in Karachi is primarily associated with PWIDs, with an HIV prevalence of 48.7% [11]. Moreover, Karachi is also experiencing an increase in the prevalence of HIV among women and children [17]. 

In this study, we conducted a comprehensive genetic and drug resistance analysis using samples obtained from ART-naïve and ART-experienced individuals living with HIV in Karachi, Pakistan. Analysis of DRMs associated with protease (PIs) and reverse transcriptase inhibitors (RTIs) remains crucial for surveillance and monitoring of drug resistance patterns and primarily to optimize alternate ART regimens, which can ensure viral suppression in PLHIV who may not be eligible for a switch to or initiation of dolutegravir (DTG) therapy because of factors such as hyperglycemia or diabetes, etc. [13,18].

## 2. Materials and Methods

### 2.1. Sample Collection 

A total of 364 HIV-positive individuals were recruited, and demographic information, including age, sex, HIV diagnosis, ART status, CD4 count, and risk factors, was collected from each individual. All samples were collected from Aga Khan University, Karachi, and Bridge Consultant Foundation, Karachi, Pakistan. Written informed consent was obtained from all study participants before carrying out any study procedures. This study was approved by the Institutional Ethical Review Committee of the School of Medicine, Nankai University, National Center for AIDS/STD Control and Prevention, China CDC, and Aga Khan University Karachi, Pakistan (4189-BBS-ERC-16). All experiments were performed in accordance with approved guidelines and regulations. 

### 2.2. DNA Extraction and HIV-pol Gene Amplification and Sequencing

Proviral DNA was extracted from each blood sample using the Qiagen (Hilden, Germany) QIAamp blood mini kit and kept at −80 °C until further use. The HIV-1 *pol* gene (complete protease and partial reverse transcriptase regions) was amplified and sequenced using a previously published protocol [19,20]. Briefly, a two-step nested PCR was performed. The first round of PCR was conducted under the following cycling conditions: PCR activation at 94 °C for 2 min, 35 cycles of denaturation at 94 °C for 15 s, annealing at 50 °C for 20 s, extension at 72 °C for 2 min, and a final extension at 72 °C for 10 min. Subsequently, a 2 μL template of first-round PCR was added to the second round of PCR with cycling conditions: PCR activation at 94 °C for 4 min, 35 cycles of denaturation at 94 °C for 15 s, annealing at 55 °C for 20 s, extension at 72 °C for 2 min, and final extension at 72 °C for 10 min. PCR amplicons were purified and sequenced using the Sanger sequencing platform (Tianyi-Huiyuan Biotech. Co., Ltd. Beijing, China). 

### 2.3. Phylogenetic and Transmission Cluster Analysis

HIV-1 pol (PR/RT) sequences, obtained by Sanger sequencing, were analyzed and edited using Sequencher v5.4.6, and the sequence quality was accessed using the WHO HIVDR QC tool (https://sequenceqc.bccfe.ca/who_qc). Multiple sequence alignment was performed using MAFFT v7 [21]. The alignment length was 1063 bp, relative to the HXB2 nucleotide position 2253–3315, which covered the entire protease and partial reverse transcriptase region. 

Initially, HIV-1 subtyping was performed using the REGA v3.0 [22] and COMET [23] subtyping tools. Subsequently, all *pol* sequences were aligned using MAFFT v7 [21] with the recent (2020) HIV-1 M group (subtype and recombinants) reference sequence alignments retrieved from the HIV LANL database (https://www.hiv.lanl.gov/content/index). The resulting alignment was used to construct a maximum-likelihood (ML) phylogenetic tree using IQ-Tree [24] with a general time-reversible plus gamma (GTR + G) nucleotide substitution model and 1000 bootstrap values and visualized in FigTree v1.4.4.

The *pol* gene ML phylogenetic tree was used to identify the transmission clusters. Transmission clusters were identified using ClusterPicker v1.2.3 [25] with an SH-aLRT support value of ≥90% and a pairwise genetic distance threshold of 3.0%. Monophyletic clusters were defined based on clades with SH-aLRT support values of ≥90% and intra-cluster genetic distance of <3.0%. 

### 2.4. HIV-1 Drug Resistance Mutation Analysis

For DRM analysis, HIV-1 protease and reverse transcriptase sequences were divided into three groups: ART-naïve, ART-experienced, and unknown ART status. DRM associated with resistance to protease and reverse transcriptase inhibitors were determined using the Stanford University HIV drug resistance database program v9.4 (https://hivdb.stanford.edu/), in accordance with the 2019 Update of Drug Resistance Mutations in HIV-1 mutation list of the International AIDS Society United States [26]. The Stanford HIV drug resistance database defines five drug resistance levels: a score between 0 and 9 denotes “susceptible”, a score between 10 and 14 denotes “potential low-level resistance”, a score between 15 and 29 denotes “low-level resistance”, a score between 30 and 59 denotes “intermediate resistance”, and a score ≥ 60 denotes “high-level resistance.”

### 2.5. Genomic Variability and Selection Pressure on DRM Sites

The variability of the *pol* sequences in the three study groups (ART-naïve, ART-experienced, and unknown ART status) was analyzed using the Shannon Entropy tool [13]. To identify the DRM sites under selection, HIV-1 protease and reverse transcriptase sequences of each group were analyzed using the Fast Unconstrained Bayesian Approximation (FUBAR) method implemented in the DATAMONKEY server (https://ww.datamonkey.org). All estimations were performed using the MG94xREV codon substitution model (https://doi.org/10.1093/molbev/msi105). A cut-off *p*-value < 0.5 was selected to classify codon sites exhibiting positive or negative selection. 

## 3. Results

### 3.1. Demographic Characteristics of the Study Individuals 

Of the 364 samples, 268 (73.6%) were successfully amplified and sequenced. Out of 268 sequences, 163 were from ART-experienced, 37 were from ART-naïve, and 68 were from individuals with unknown ART status. The remaining 96 (26.4%) amplification failures were possibly due to low viral loads or genetic diversity attributed to quasispecies in HIV-infected individuals [27,28]. Of the 268 individuals, 88% were male, and 11.2% were female of Pakistani origin. The median age of the infected individuals was 36 years (interquartile range (IQR) 30–40 years). The highest number of individuals was diagnosed in 2021 (108, 40.2%), followed by 2015 (26, 9.7%). The demographic characteristics of the participants are presented in (Table 1). 

### 3.2. Distribution of HIV-1 Genetic Diversity

**Figure 1:**
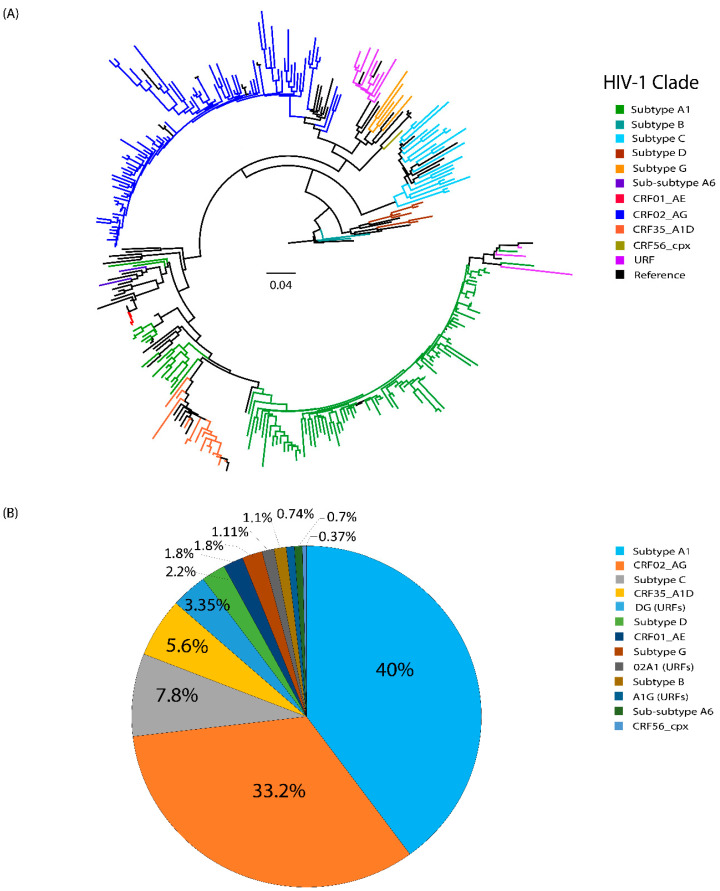
HIV-1 subtype distribution in Karachi. (**A**) Maximum likelihood (ML) phylogenetic tree consisting of 268 *pol* sequences amplified from PLHIV and 50 HIV-subtype reference sequences (colored black). Tree branches are colored according to subtype. The scale bar at the center indicates the number of substitutions per site along the tree branches. (**B**) The pie chart represents the proportion of subtypes in the study groups.

Maximum likelihood phylogenetic analysis of 268 partial *pol* (complete protease: 1–99 codon and partial reverse transcriptase: 1–255 codon) sequences revealed 13 distinct HIV-1 strains circulating in Karachi (Figure 1A). Among them, subtype A1 was the most common strain (n = 107, 40%), followed by CRF02_AG (n= 89, 33.2%) and subtype C (n= 21, 7.8%). In addition, 14 possible recombinants (5.2%) were also detected. Further phylogenetic and recombination analyses of these 14 sequences showed that 9 (3.3%) were DG, 3 (1.1%) were 02A1, and 2 (0.7%) were A1G unique recombinant forms (URFs) (Figure 1B). Interestingly, two sub-subtype A6 strains were detected in Karachi for the first time. 

Comparative HIV-1 subtype analysis among the three study groups showed that CRF02_AG was the dominant clade in the ART-experienced group (25.7%), subtype A1 in ART-naïve (5.6%) and unknown ART status groups (11.9%) (Table 2). 

### 3.3. Prevalence and Patterns of HIV Drug Resistance Mutations 

The overall prevalence of HIV DRMs was 47.76% (128/268). Among the ART-naïve and ART-experienced groups, DRM prevalence was 5.6% and 30.6%, respectively, whereas 11.5% of the ART unknown-status group harbored DRM. 

**Figure 2:**
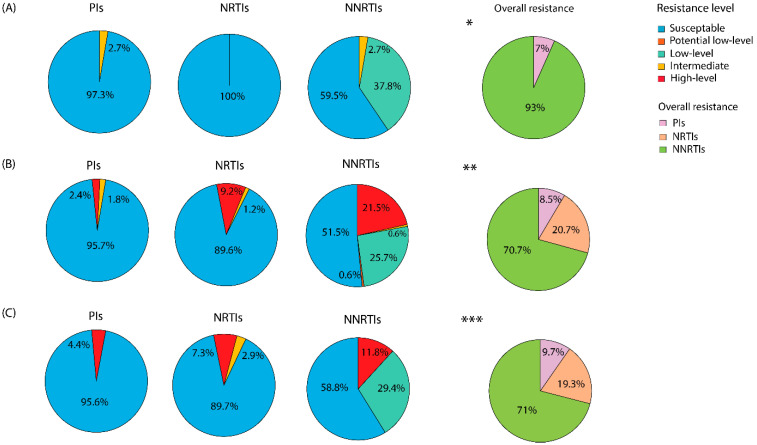
Distribution of antiretroviral drug resistance among the study participants. Predicted resistance levels were based on Stanford HIV drug resistance scores. Scores < 10 indicate susceptibility, scores of 10–14 indicate potential low-level resistance, scores of 15–29 indicate low-level resistance, scores of 30–59 indicate intermediate resistance, and scores ≥ 60 indicate high-level resistance. Resistance to antiretroviral drugs among (**A**) ART-naïve group, (**B**) ART-experienced group, and (**C**) unknown ART status group individuals. Overall drug resistance against any antiretroviral class among the different groups is indicated as follows: * ART-naïve, ** ART-experienced, *** unknown status individuals. PIs, protease inhibitors, NRTIs, nucleoside reverse transcriptase inhibitors, NNRTIs, and non-nucleoside reverse transcriptase inhibitors.

The ART-naïve group was susceptible to NRTIs, whereas resistance to NNRTIs and PIs was 93% and 7%, respectively. Among ART-naïve individuals with DRMs, 2.7% and 2.7% harbored intermediate-level resistance (Stanford HIV drug resistance score of 30–59) against PIs and NNRTIs, respectively (Figure 2A).

In the ART-experienced group, DRMs against NNRTIs were considerably high (70.7%), followed by NRTIs (20.7%) and PIs (8.5%). Of these, 2.4%, 9.2%, and 21.8% harbored high-level resistance (Stanford HIV drug resistance score of ≥60) against PIs, NRTIs, and NNRTIs, respectively (Figure 2B). Similarly, among the individuals in the unknown ART status group, DRMs against NNRTIs remained high (71%), followed by NRTIs (19.3%) and PIs (9.7%). Moreover, 4.4%, 7.3%, and 11.8% harbored high-level resistance (Stanford HIV drug resistance score of ≥60) against PIs, NRTIs, and NNRTIs, respectively (Figure 2C). 

Among the 3 study groups, 43 distinct DRMs were detected that were classified as major or minor mutations by the International AIDS Society report and showed resistance to different antiretroviral drugs (Table 3). Comparative analysis of DRMs in the 3 study groups showed a high prevalence of DRMs such as E138A (33.7%), K103N (12.2%), V179E (3.0%), and Y188L (2.4%), which are associated with resistance against NNRTIs; DRM M184V (6.7%), M184I (2.4%), K219E (2.4%), D67N (1.8%), K65R (1.8%), and K70R (1.8%), which are associated with resistance to NRTIs in the ART-experienced group (Table 3). Moreover, 5 major DRMs, T69D (1.2%), and Y115F (1.2%) were associated with resistance to NRTIs, and K103S (1.2%), H221Y (1.2%), and V106M (1.2%), associated with resistance against NNRTIs, were observed in three sequences only (Table 3). Additionally, a minor mutation, L10F (2.4%), and a minor/major mutation, M46I (1.2%), associated with resistance to PIs, were observed in 4 and 2 sequences, respectively (Table 3). 

Similarly, DRM analysis of the ART-naïve group showed a high prevalence of the mutation E138A (37.8%) associated with resistance to NNRTIs. Similarly, DRMs E138G (2.7%) and L10F (2.7%) were observed in 1 sequence each and were associated with resistance to NNRTI and PIs (Table 3). Analysis of DRMs in the unknown ART group showed a high prevalence of DRMs such as E138A (35.3%), K103N (5.8%), and V179T (2.9%), associated with resistance to NNRTIs, and M184V (5.8%), M41L (2.9%), K65R (2.9%) and Y115Y (2.9%), associated with resistance to NRTIs (Table 3). 

**Figure 3:**
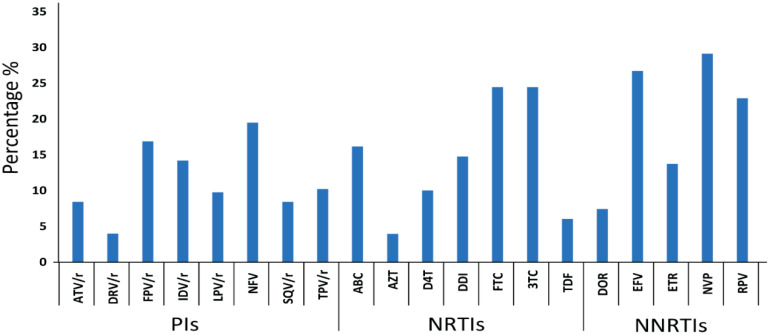
Prevalence of drug resistance against PIs, NRTIs, and NNRTIs. Antiretroviral drugs abbreviations: PIs. ATV/r, Atazanavir/ritonavir; DRV/r, darunavir/ ritonavir; FPV/r, fosamprenavir/ritonavir; IDV/r, indinavir/ritonavir; LPV/r, lopinavir/ritonavir; TPV/r, tipranavir/ritonavir; NFV, nelfinavir; SQV/r, saquinavir/ritonavir. NRTIs. ABC, abacavir, AZT, zidovudine; D4T, stavudine; DDI, didanosine; FTC, emtricitabine; 3TC, lamivudine; TDF, tenofovir. NNRTIs. DOR, doravirine; EFV, efavirenz; ETR, etravirine; NVP, nevirapine; RPV, rilpivirine.

Similarly, analysis of DRM on ART efficacy showed that DRMS mainly affected nevirapine (29.1%), efavirenz (26.7%), and rilpivirine (23%) among NNRTIs; emtricitabine (24%) and 3TC lamivudine (24%) among NRTIs; and nelfinavir (20%), fosamprenavir/ritonavir (16.8%), and IDV/r indinavir/ritonavir (14.2%) among PIs (Figure 3).

### 3.4. DRM-Associated Transmission Clusters Analysis

**Figure 4:**
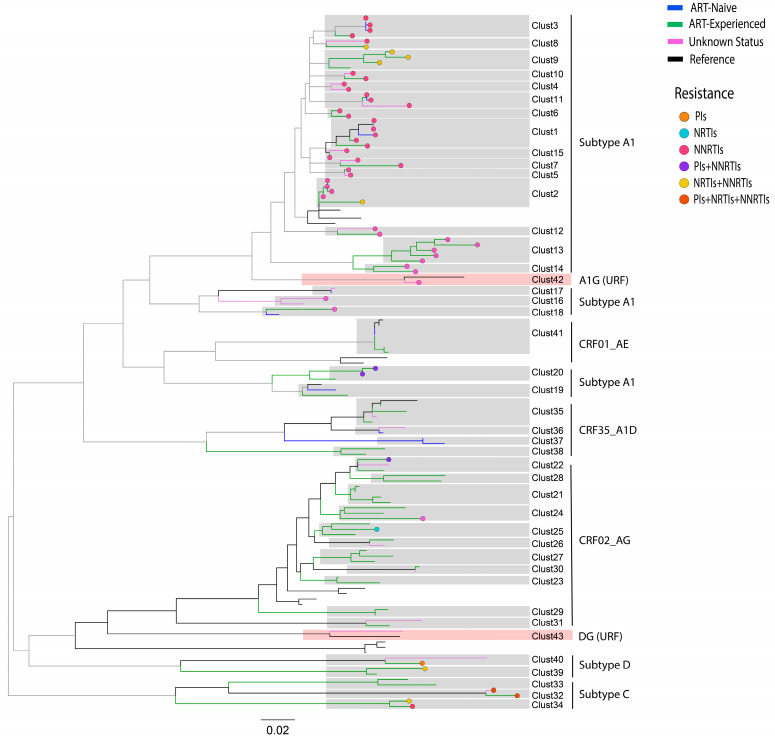
Maximum-likelihood phylogenetic tree indicating relationships between transmission clusters and mutations among the three study groups. Tree branches are colored according to the following scheme: green: ART-experienced; blue: ART-naïve; lavender: unknown status; and black: HIV-1 subtypes reference sequences. Tree branch tips are colored to indicate resistance against different antiretroviral drugs: orange: protease inhibitors (PIs); light blue: nucleoside reverse transcriptase inhibitors (NRTIs); rose: non-nucleoside reverse transcriptase inhibitors (NNRTIs); purple; PIs+NNRTIs; yellow; NRTIs+NNRTIs; dark; PIs+NRTIs+NNRTIs. The scale bar at the center indicates the number of substitutions per site along the tree branches. The two transmission clusters, URF DG and A1G, with reference sequences, are highlighted in light purple.

Out of 268 sequences, 110 (41%) were segregated into 43 transmission clusters (sizes ranging from 2 to 7 sequences). The 43 transmission clusters included 29 dyads (consisting of two sequences) and 14 networks (composed of 3–7 sequences) (Figure 4). 

Among the 43 transmission clusters, clusters 1–20 comprised subtype A1, clusters 21–31 comprised CRF02_AG, clusters 32–34 comprised subtype C, clusters 35–38 comprised CRF35_A1D, and clusters 39–40 comprised subtype D. Meanwhile, clusters 41, 42, and 43 comprised CRF01_AE, URF A1G, and DG, respectively (Table 4). 

The analysis of DRMs in the 110 individual sequences distributed throughout the 43 transmission clusters showed that 57 sequences harboring DRMs were segregated into 26 transmission clusters (Figure 4). Among the 26 transmission clusters, 18 clusters comprised subtype A1, 3 clusters comprised CRF02_AG, 2 clusters comprised subtype C and subtype D, and 1 cluster comprised URF A1G (Table 4). 

Comparative analysis of DRMs between the study groups showed that 39 sequences belonged to the ART-experienced group, 4 sequences belonged to the ART-naïve group, and 14 sequences belonged to the unknown status group (Figure 4). The most common DRM was E138A, which was found in 43 sequences across the three study groups, including 28 sequences belonging to the ART-experienced group, 11 sequences belonging to the unknown status group, and 4 sequences belonging to the ART-naïve group (Table 4). Similarly, DRM E138A was shared among 23 sequences, including 11 in the ART-experienced group, 4 in the ART-naïve group, and 8 in the unknown ART status group. Interestingly, all 23 sequences harboring the E138A mutation were distributed across nine subtype A1 clusters (Clust3, Clust8, Clust10, Clust11, Clust1, Clust15, Clust7, Clust5, and Clust12) (Table 4 and Figure 4). DRM L10F was found in two sequences within the subtype A1 cluster (Clust20), with both sequences belonging to the ART-experienced group. DRM K103N was found in two sequences within the subtype C cluster (Clust34), with both sequences belonging to the ART-experienced group. Similarly, DRMs D67N, T69D, K70R, M184V, T215I, K219E, V106M, and Y188L were found in two sequences within the subtype C cluster (Clust32), with each sequence belonging to the ART-experienced and unknown status groups (Table 4) (Figure 4). 

### 3.5. Genomic Variability and Selection Pressure on DRM Sites

Next, a comparative analysis of genomic variability and selection pressure on DRM sites in the three study groups was performed. The analysis showed that the four DRM sites in the ART-naïve group (10, 138, 179, and 230) had a high entropy score, which was greater than the ART-naïve group’s mean entropy value (0.10). Similarly, 11 DRM sites in the ART-experienced group (10, 41, 70, 101, 103, 106, 138, 179, 184, 221, and 230) showed high entropy scores, which were higher than the mean entropy value (0.12) in the ART-experienced group. A total of 9 DRM sites in the unknown ART status group (10, 69, 101, 103, 138, 179, 184, 190, and 230) exhibited high entropy scores, which were higher than the mean entropy value (0.13) in the unknown status group (Supplementary Table S1).

The selection pressure predicted using the FUBAR method performed separately for each group indicated four distinct DRM sites under positive selection pressure, including DRM site 138 in the ART-naïve group, DRM site 188 in the ART-experienced group, and DRM sites 103 and 115 in the unknown status group, while all other DRM sites were under negative selection. Further analysis of DRM sites between the 3 groups showed DRM sites 69, 101, 179, 184, and 190 (−0.99, −0.95, −0.97, −0.93, and −0.99, respectively) to be under high negative selection pressure in the ART-naïve group; DRM sites 10, 30, 41, 54, 65, 100, 101, 190, 221, and 230 (−1.00, −1.00, −0.94, −0.99, −0.97, −0.99, −0.87, −0.99, −0.95, and −0.93, respectively) to be under high negative selection pressure in the ART-experienced group; while DRM sites 46, 47, 54, 106, and 138 (−0.95, −0.90, −0.99, −0.99, and −0.98, respectively) to be under high negative selection pressure in the unknown status group. Moreover, DRM site 103 was observed under positive selection in the ART-experienced group (Supplementary Table S1). 

## 4. Discussion

In this study, we analyzed 268 HIV-1 *pol* sequences from people living with HIV (PLHIV) in Karachi, Pakistan. We aimed to identify drug resistance mutations, quantify the levels of drug resistance, and determine the distribution of HIV-1 genetic diversity in Karachi City. The study participants were divided into 3 groups based on their ART history (ART-experienced n = 168, ART-naïve n = 37, and unknown status n = 68). In our study, 88% of PLHIV were male, and 61.9% were people who inject drugs (PWIDs). This finding is consistent with previous studies showing that male PWIDs comprise the predominant key group [13,17]. However, key populations, including MSM (3.3%) and FSW (1.1%), were underrepresented in our dataset. Similarly, 33.5% of the study participants recorded no associated risk factors (Table 1).

Among the study participants, 40% were infected with HIV-1 subtype A1, 33.2% with CRF02_AG, and 7.8% with subtype C (Figure 1B). In Pakistan, subtype A1 continues to be the predominant clade [8,13,14], although the proportion of CRF02_AG is gradually increasing [7,29]; and subtype C and CRF35_A1D have also been observed in many Pakistani PLHIV [13,29]. We also observed sub-subtype A6 for the first time in Pakistan, which is the predominant strain in Former Soviet Union countries [30]. Such an increase in HIV-1 subtype composition may be attributed to people immigrating from regions where these strains are highly prevalent [31]. Similarly, we identified 14 distinct unique recombinant forms, each of which was identified for the first time in Karachi, Pakistan, which can further increase HIV-1 diversity in the region [32,33]. In high-risk groups, such as PWIDs and FSWs, which have a high possibility of getting co-infections with different subtypes, the chances of the emergence of new recombinant forms are high [31,34]. 

The overall prevalence of DRMs in our study was 47.76%. The proportion of transmitted NNRTIs and PIs resistance was 93% and 7%, while NRTIs remained susceptible. The proportion of acquired NNRTIs, NRTIs, and PIs resistance was 70.7%, 20.7%, and 8.5%, respectively. A comparative analysis of DRMs in the three study groups showed the presence of multiple DRMs associated with resistance to reverse transcriptase inhibitors. The DRM E138A, which can confer major resistance against rilpivirine (PRV) [14,30] was particularly high among the three study groups (Table 3). Previous studies have also detected the E138A mutation among subtype A1 sequences of Pakistani PLHIV, reporting varying prevalence in ART-naïve (8–39%) and ART-experienced individuals [14,29]. The E138A mutation most frequently occurs in subtype C and is associated with reduced RPV susceptibility by 2.9-fold [35]. A high prevalence of E138A has also been reported in PLHIV from Mozambique (12%) [36], China (14.3%) [37], and Greece (7.7%) [38]. K103N was the second most prevalent DRM identified among ART-experienced and unknown status group individuals, which confers significant resistance to efavirenz and nevirapine [39]. Furthermore, DRM M184I identified among ART-experienced and unknown status groups can confer major resistance against abacavir, emtricitabine, and lamivudine (Table 3). The presence of DRM associated with resistance to efavirenz and lamivudine, which are part of the ART regimen administered to PLHIV in Pakistan, may lead to ART failure [13]. DRMs K103N and M184V have been frequently reported in Pakistani PLHIV [13,14,29,40]. Similarly, we observed high-level resistance against NNRTIs among the ART-experienced group (21.5%) and the unknown status group (11.8%). In both the ART-experienced and unknown status groups, the DRMs E138A and K103N showed high-level resistance against NNRTIs, including EFV, NVP, and RPV. K103N is the most commonly transmitted DRM, associated with virological failure [41], and can decrease NVP and EFV susceptibility by 50- and 20-fold, respectively [42,43]. 

A considerable number of individuals in the three groups carrying DRMs (51.8%) were observed in the transmission clusters, confirming the transmission of DRMs in the region. The most common DRM, E138A, was observed in 18 of 43 transmission clusters (Figure 4); DRM E138A was previously detected in transmission clusters of subtypes A1 and CRF02_AG among ART-naïve children in Larkana, Pakistan [29]. The prevalence of NRTI-associated DRMs in ART-experienced patients in Pakistan (20.7%) is consistent with findings from other countries. For example, studies from India and China reported NRTI-associated DRMs in 44.5% and 74.4% of ART-experienced PLHIVs [44,45]. Despite the high prevalence of NRTI mutations in ART-experienced patients, the transmission of these mutations appears to be relatively low. This may be due to the fitness cost associated with certain DRMs against NRTIs, such as DRM M184V, which can significantly reduce viral replication capacity. Consequently, values with NRTI DRMs may be less transmissible [46,47].

Shannon entropy analysis revealed that DRM sites 10, 138, 179, and 230 in the three study groups exhibited higher entropy than mean entropy (Supplementary Table S1). High entropy at different codon positions can correlate with an increased probability of mutations [48]. This finding is consistent with our previous study, in which high Shannon entropy was observed in the HIV-1 *pol* gene of ART-naïve individuals in Pakistan [13]. In our study, DRM sites 138, 188, 103, and 115 were under positive selection pressures (Supplementary Table S1). The prevalence of DRM at sites 138 and 103 was frequently observed in all 3 study groups. Positive selection of pol-encoded amino acids in PLHIV experiencing virological failure can potentially alter the structural and biological functions of the viral proteins. This adaptive process may serve as a mechanism by which the virus evades the pressure exerted by antiretroviral drugs [49]. 

The limitations of this study include the absence of demographic and clinical data of PLHIV from whom *pol* sequences were obtained. These data will enable us to establish an association between DRM and disease progression. Additionally, the small sample size may not accurately represent the overall status of HIV-1 among the key populations in Karachi. The lack of HIV integrase gene sequencing is because none of the individuals were on integrase inhibitors at the time of sample collection. In the future, larger samples from key populations in Karachi could provide a clearer picture of drug-resistance mutations and transmission linkages among Pakistani PLHIV, especially those living in Karachi. 

## 5. Conclusions

In conclusion, the complex genetic diversity of HIV-1 and the high prevalence of DRMs in ART-experienced patients are concerning, which can lead to the failure of first-line drugs, particularly in regions such as Pakistan, where second-line drugs are not readily available. This study emphasizes the importance of continuous HIV drug resistance surveillance to support evidence-based HIV prevention, precise ART, and targeted AIDS care.

## Figures and Tables

**Table 1:** Demographic characteristics of study populations.

Characteristic	
Participants	
Total number of participants	268
ART-Experienced n (%)	163 (60.8)
ART-Naïve n (%)	37 (13.8)
Unknown status n (%)	68 (25.3)
Sex	
Male, n (%)	236 (88)
Female, n (%)	30 (11.2)
No record, n (%)	2 (0.74)
Age (years), median (IQR)	36 (30–40)
CD4 count (cells/mm^3^), median (IQR)	429 (422–490)
ART regimen	
TDF + 3TC + EFV, n (%)	163 (60.8)
Year of diagnosis	
1995–2010 n (%)	45 (16.8)
2011–2022 n (%)	219 (82)
No record n (%)	4 (1.5%)
Risk factor	
PWID n (%)	166 (61.9)
MSM n (%)	9 (3.35)
FSW n (%)	3 (1.1)
No record n (%)	90 (33.5)

Abbreviations: ART, antiretroviral therapy; TDF, tenofovir disoproxil fumarate; 3TC, lamivudine; EFV, efavirenz; PWID, People who inject drugs; MSM, men who have sex with men; FSW, female sex worker.

**Table 2:** HIV-1 subtype diversity among the study populations.

Subtype	Total n (%)	ART-Experienced	ART-Naïve	Unknown ART Status
Subtype A1	107 (40)	60 (22.3)	15 (5.6)	32 (11.9)
CRF02_AG	89 (33.2)	69 (25.7)	6 (2.2)	14 (5.2)
Subtype C	21 (7.8)	12 (4.5)	3 (1.1)	6 (2.2)
CRF35_A1D	15 (5.6)	4 (1.86)	4 (1.5)	7 (2.6)
DG (URFs)	9 (3.35)	3 (1.1)	4 (1.5)	2 (0.7)
Subtype D	6 (2.2)	4 (1.5)	0 (0)	2 (0.7)
CRF01_AE	5 (1.86)	3 (1.1)	1 (0.37)	1 (0.37)
Subtype G	5 (1.86)	1 (0.4)	2 (0.74)	2 (0.7)
02A1 (URFs)	3 (1.1)	2 (0.74)	1 (0.37)	0 (0)
Subtype B	3 (1.1)	3 (1.1)	0 (0)	0 (0)
A1G (URFs)	2 (0.7)	1 (0.4)	0 (0)	1 (0.37)
Sub-subtype A6	2 (0.7)	1 (0.4)	1 (0.37)	0 (0)
CRF56_cpx	1 (0.37)	0 (0)	0 (0)	1 (0.37)

Abbreviation: ART, antiretroviral therapy; URF, unique recombinant form.

**Table 3:** Classification of the drug resistance mutations DRMs.

Drugs	Mutation	Naïve, N (%) n = 37	Experienced, N (%) n = 163	Unknown Status, N (%) n = 68	Mutation Classification	Drugs Affected by the DRMs
**PIs**	* M46I	0 (0)	2 (1.2)	0 (0)	Minor/Major	FPV/r, * IDV/r
	* I54V	0 (0)	1 (0.6)	0 (0)	Major	* FPV/r, * IDV/r
	* D30N	0 (0)	0 (0)	1 (1.5)	Major	TPV/r
	* I47V	0 (0)	1 (0.6)	0 (0)	Minor/Major	FPV/r, NFV, * TPV/r
	L10F	1 (2.7)	4 (2.4)	1 (1.5)	Minor	FPV/r, IDV/r, NFV
	L33F	0 (0)	1 (0.6)	0 (0)	Minor	FPV/r, IDV/r, NFV
	G73S	0 (0)	1 (0.6)	1 (1.5)	Minor	FPV/r, IDV/r, NFV, SQV/r
	G48R	0 (0)	1 (0.6)	1 (1.5)	Minor	FPV/r, IDV/r, NFV
**NRTIs**	* M41L	0 (0)	0 (0)	2 (2.9)	TAM, Major	* ABC, * AZT, * D4T, * DDI, * FTC, * 3TC, * TDF
	* M184I	0 (0)	4 (2.4)	1 (1.5)	Major	* ABC, * FTC, DDI, * 3TC
	* M184V	0 (0)	11 (6.7)	4 (5.8)	Major	* ABC, * FTC, * 3TC
	* A62V	0 (0)	1 (0.6)	0 (0)	TAM, Major	* ABC, * DDI, * FTC, * 3TC
	* K65R	0 (0)	3 (1.8)	2 (2.9)	Major	* ABC, DDI, * FTC, * 3TC
	* D67N	0 (0)	3 (1.8)	1 (1.5)	TAM, Major	* ABC, * AZT, * D4T, * DDI, FTC, 3TC
	* K70R	0 (0)	3 (1.8)	0 (0)	TAM, Major	* ABC, * AZT, * D4T, * DDI
	* K70E	0 (0)	1 (0.6)	0 (0)	Major	ABC, D4T, DDI, FTC, 3TC, * TDF
	* T215Y	0 (0)	2 (1.2)	0 (0)	TAM, Major	* ABC, * AZT, * D4T, * DDI, FTC, 3TC
	* K219E	0 (0)	4 (2.4)	0 (0)	TAM, Major	* ABC, * AZT, * D4T, * DDI, FTC, 3TC
	* T69D	0 (0)	2 (1.2)	1 (1.5)	Major	* AZT, * D4T, * DDI, * FTC, * 3TC
	T215I	0 (0)	2 (1.2)	0 (0)	Minor	AZT, D4T, DDI, FTC, 3TC
	* Y115F	0 (0)	2 (1.2)	2 (2.9)	Major	ABC, DDI, FTC, 3TC
**NNRTIs**	* K103N	0 (0)	20 (12.2)	4 (5.8)	Major	* EFV, * NVP
	* K103S	0 (0)	2 (1.2)	0 (0)	Major	* EFV, * NVP
	* E138A	14 (37.8)	55 (33.7)	24 (35.3)	Minor/Major	ETR, * RPV
	* E138G	1 (2.7)	0 (0)	0 (0)	Minor/Major	* RPV
	V179E	0 (0)	5 (3.0)	0 (0)	Minor	EFV, NVP, RPV
	V179T	0 (0)	1 (0.6)	2 (2.9)	Minor	EFV, NVP, RPV
	V179D	0 (0)	1 (0.6)	1 (1.5)	Minor	EFV, NVP, RPV
	* V179L	0 (0)	2 (1.2)	0 (0)	Minor/Major	EFV, ETR, NVP, * RPV
	* M230I	0 (0)	3 (1.8)	1 (1.5)	Minor/Major	NVP, * RPV
	* Y188L	0 (0)	4 (2.4)	0 (0)	Major	* DOR, * EFV, * NVP, * RPV
	* H221Y	0 (0)	2 (1.2)	0 (0)	Major	EFV, ETR, NVP, * RPV
	G190S	0 (0)	0 (0)	1 (1.5)	Minor	NVP, RPV
	* G190A	0 (0)	2 (1.2)	0 (0)	Minor/Major	* EFV, * NVP, RPV
	G190E	0 (0)	0 (0)	1 (1.5)	Minor	DOR, EFV, ETR, NVP, RPV
	* V106M	0 (0)	2 (1.2)	1 (1.5)	Major	DOR, * EFV, * NVP, RPV
	V106I	0 (0)	1 (0.6)	1 (1.5)	Minor	DOR, EFV, NVP, RPV
	* Y181C	0 (0)	1 (0.6)	1 (1.5)	Major	* EFV, * ETR, * NVP, RPV
	* P225H	0 (0)	1 (0.6)	1 (1.5)	Major	* EFV, NVP
	P236L	0 (0)	1 (0.6)	0 (0)	Minor	DOR
	* L100I	0 (0)	1 (0.6)	1 (1.5)	Major	DOR, * EFV, * ETR, * NVP, * RPV
	K101H	0 (0)	1 (0.6)	0 (0)	Minor	EFV, ETR, NVP, RPV
	K101E	0 (0)	1 (0.6)	0 (0)	Minor	DOR, EFV, NVP, RPV

Abbreviations: PI, protease inhibitor; NRTI, nucleoside reverse transcriptase inhibitor; NNRTI, non-nucleoside reverse transcriptase inhibitor; TAM, thymidine analogue-associated mutations. The mutation classification column represents mutations classified as either minor or major. The second and last columns indicate the association of a mutation with resistance to a particular drug, as presented in the Stanford drug resistance database. * and bold mutations and drug names indicate major resistance to a particular drug, as identified by the International AIDS Society (IAS) 2019 report.

**Table 4:** Number of transmission clusters identified in the phylogenetic tree with shared drug resistance mutations.

Cluster Name	HIV-1 Subtype	Number of Nodes	Risk Factor	ART History	Number with Shared DRM
Clust1	A1	5	PWID, N/A	Experienced, Naïve, unknown status	E138A, n = 5
Clust2	A1	5	PWID, N/A	Experienced, unknown status	E138A, n = 5, M184V, n = 1, G190A, n = 1
Clust3	A1	4	PWID, N/A	Experienced, Naïve, unknown status	E138A, n = 4
Clust4	A1	2	PWID	unknown status	E138A, n = 2
Clust5	A1	2	PWID	Experienced, unknown status	E138A, n = 2
Clust6	A1	2	PWID	Experienced	E138A, n = 2
Clust7	A1	2	N/A	Experienced, unknown status	E138A, n = 2
Clust8	A1	2	PWID	Experienced, unknown status	E138A, n = 2, K219E n = 1
Clust9	A1	4	PWID, N/A	Experienced	E138A, n = 3, K103N, n = 3, M184V, n = 3
Clust10	A1	2	PWID, N/A	Experienced, unknown status	E138A, n = 2
Clust11	A1	3	PWID, N/A	Experienced, Naïve, unknown status	E138A, n = 3, K103N, n = 1
Clust12	A1	2	PWID, N/A	Experienced, unknown status	E138A, n = 2
Clust13	A1	5	PWID, N/A	Experienced	E138A, n = 2, K103N, n = 1
Clust14	A1	2	PWID, N/A	Experienced	E138A, n = 2
Clust15	A1	2	PWID	Experienced	E138A, n = 2, L10F, n = 1
Clust16	A1	2	PWID, N/A	unknown status	K103N, n = 1
Clust17	A1	2	PWID, N/A	Naïve, unknown status	
Clust18	A1	2	N/A	Experienced, Naïve	M230I, n = 1
Clust19	A1	2	N/A	Experienced, Naïve	
Clust20	A1	3	PWID	Experienced, Naïve	E138A, n = 2 L10F, n = 2
Clust21	CRF02_AG	4	PWID, N/A	Experienced	
Clust22	CRF02_AG	3	PWID, N/A	Experienced, unknown status	G48R, n = 1, M230I, n = 1
Clust23	CRF02_AG	2	PWID, N/A	Experienced	
Clust24	CRF02_AG	3	PWID, N/A	Experienced	E138A, n = 1
Clust25	CRF02_AG	3	PWID	Experienced	T69D, n = 1
Clust26	CRF02_AG	2	PWID, N/A	Experienced, unknown status	
Clust27	CRF02_AG	3	PWID, N/A	Experienced	
Clust28	CRF02_AG	2	N/A	Experienced	
Clust29	CRF02_AG	2	PWID	Experienced	
Clust30	CRF02_AG	2	N/A, MSM	Experienced, unknown status	
Clust31	CRF02_AG	2	PWID	Experienced, unknown status	
Clust32	C	2	N/A	Experienced, unknown status	D67N n = 2, T69D n = 2, K70R n = 2, M184V n = 2, T215I n = 2, K219E n = 2, V106M n = 2, Y188L n = 2
Clust33	C	2	PWID, FSW	Experienced	
Clust34	C	2	PWID, FSW	Experienced	K103N, n = 2, M184V, n = 1
Clust35	CRF35_A1D	5	PWID, N/A	Experienced, unknown status	
Clust36	CRF35_A1D	2	PWID, N/A	Naïve, unknown status	
Clust37	CRF35_A1D	2	N/A, MSM	Naïve	
Clust38	CRF35_A1D	2	PWID	Experienced	
Clust39	D	2	PWID, N/A	Experienced	M184V, n = 1, V179D, n = 1, Y188L, n = 1
Clust40	D	2	PWID, N/A	Experienced, unknown status	M46I, n = 1
Clust41	CRF01_AE	7	PWID, N/A	Experienced, Naïve, unknown status	
Clust42	A1G	2	PWID	unknown status	E138A, n = 1
Clust43	DG	2	N/A	unknown status	

Abbreviations: ART, antiretroviral therapy; PWID, people who inject drugs; MSM, men who have sex with men; FSW, Female sex worker; N/A, not available.

## Data Availability

The sequences analyzed in the study can be found in online repositories (https://www.ncbi.nlm.nih.gov/genbank/) with the following NCBI accession numbers: PP171667-PP171703, and PP178710-PP178940.

## Supplementary Materials

The following supporting information can be downloaded at https://www.mdpi.com/article/10.3390/v16060962/s1. Table S1. Genetic variability and selection pressure on DRM sites.
