# Supplementary material for: Human Immunodeficiency Virus Type-1 Genetic Diversity and Drugs Resistance Mutations among People Living with HIV in Karachi, Pakistan
Source: Viruses. 2024 Jun 14;16(6):962. doi: 10.3390/v16060962 (PMC11209141; doi:10.3390/v16060962)
Supplement: Supplementary file 1 [file viruses-16-00962-s001.zip › viruses-3027874-supplementary.pdf]

Table S1. Genetic variability and selection pressure on DRM sites.

**Drugs**	**Mutations**	**Entropy**			**Selection pressure**		
		**Naïve**	**Experienced**	**unknown status**	**Naïve**	**Experienced**	**unknown status**
**PIs**	M46I	0.00	0.06	0.00	-0.93	-0.92	-0.95
	I54V	0.00	0.03	0.00	-0.88	-0.99	-0.99
	D30N	0.00	0.00	0.07	-0.97	-1.00	-0.64
	I47V	0.00	0.03	0.00	-0.87	-0.79	-0.90
	L10F	**0.33**	**0.40**	**0.38**	0.57	-1.00	-0.99
	L33F	0.00	0.07	0.00	-0.84	-0.81	-0.82
	G73S	0.00	0.03	0.07	-0.99	-0.99	-0.99
	G48R	0.00	0.03	0.07	-0.87	-0.99	-0.96
**NRTIs**	M184I<br>M184V	0.00	**0.34**	**0.29**	-0.93	-0.24	-0.47
	M41L	0.00	**0.15**	0.07	-0.93	-0.94	-0.90
	A62V	0.00	0.03	0.00	-0.99	-1.00	-1.00
	K65R	0.00	0.09	0.13	-0.95	-0.97	-0.90
	D67N	0.00	0.09	0.07	-0.99	-1.00	-1.00
	K70R<br>K70E	0.00	**0.13**	0.07	-0.95	-0.99	-0.99
	T215Y<br>T215I	0.00	0.10	0.00	-0.99	-0.99	-1.00
	K219E	0.00	0.11	0.00	-0.99	-1.00	-0.98
	T69D	0.00	0.10	**0.15**	-0.99	-0.96	-0.95
	Y115F	0.00	0.03	0.13	-0.99	-1.00	**0.53**
**NNRTIs**	K103N<br>K103S	0.00	0.51	0.29	-0.95	0.11	**0.57**
	E138A<br>E138G	0.77	**0.63**	**0.64**	**0.60**	-0.89	-0.98
	V179E<br>V179T<br>V179L<br>V179D	**0.69**	**0.95**	**0.80**	0.97	-0.95	-0.78
	M230I	**0.12**	**0.30**	**0.20**	-0.62	-0.93	-0.90
	Y188L	0.00	0.11	0.07	-0.79	**0.81**	-0.57
	H221Y	0.00	**0.67**	0.00	-0.82	-0.95	-0.84
	G190S<br>G190A<br>G190E	0.00	0.10	**0.22**	-0.99	-0.99	-0.80
	V106M<br>V106I	0.00	**0.13**	0.07	-0.94	-0.96	-0.99
	Y181C	0.00	0.03	0.07	-0.99	-1.00	-0.99
	P225H	0.00	0.07	0.07	-1.00	-1.00	-1.00
	P236L	0.00	0.07	0.07	-0.99	-1.00	-1.00
	L100I	0.00	0.06	0.07	-0.84	-0.99	-0.98
	K101H<br>K101E	0.00	**0.20**	**0.15**	-0.95	-0.87	-0.65

The mean entropy value for ART-naïve was 0.10, ART-experienced was 0.12, and the unknown status group was 0.13. The entropy scores greater than the mean value for each group were characterized as high, while the entropy scores less than the mean value were characterized as low; 0 indicates no entropy. In Column 3, the high-entropy sites in each group are bold and highlighted. In column 6, DRM sites under positive selection pressure are shown in bold, while DRM sites under high negative selection are underlined.
